# Preswitch Regimens Associated With Weight Gain Among Persons With HIV who Switch to Integrase Inhibitor–Containing Regimens

**DOI:** 10.1093/ofid/ofae752

**Published:** 2025-03-21

**Authors:** Melissa Klein Cutshaw, Mahmoud Harding, Clemontina A Davenport, Nwora Lance Okeke

**Affiliations:** Division of Infectious Diseases, Duke University School of Medicine, Durham, North Carolina, USA; Department of Statistics, North Carolina State University, Raleigh, North Carolina, USA; Department of Biostatistics and Bioinformatics, Duke University School of Medicine, Durham, North Carolina, USA; Division of Infectious Diseases, Duke University School of Medicine, Durham, North Carolina, USA

**Keywords:** HIV, antiretroviral therapy, integrase inhibitors, weight gain, tenofovir

## Abstract

**Background:**

Weight gain associated with integrase strand transfer inhibitors (INSTIs) is well documented. However, recent reports suggest that the observed weight gain among persons who switch to INSTIs may be associated with their preswitch regimen.

**Methods:**

We conducted retrospective analyses of persons with HIV on antiretroviral therapy who switched to a second-generation INSTI–containing regimen (bictegravir/dolutegravir) at the Duke Adult Infectious Diseases Clinic (Durham, NC, USA) between 2014 and 2021. The outcome was weight change, operationalized as percent weight change, absolute weight change (kg), gaining ≥5% of preswitch weight, and gaining ≥10% of preswitch weight. The primary exposure was preswitch regimen.

**Results:**

Our analysis included 750 persons. Cohort demographics were as follows: mean age (SD) 51 (11) years, 30% female at birth, 58% Black, 4% Hispanic ethnicity. At regimen switch, the mean CD4 count was 701 cells/mm^3^, and 68% had a viral load ≤20 copies/cc. Persons with preswitch regimens containing efavirenz had higher odds of gaining ≥5% body weight (odds ratio [OR], 1.62, 95% CI, 1.13–2.32) and ≥10% body weight (OR, 1.68; 95% CI, 1.02–2.73) after regimen switch, adjusted for age, sex, race, ethnicity, and preswitch body mass index. Persons with preswitch regimens containing tenofovir disoproxil (TDF) also had higher odds of gaining ≥5% body weight (OR, 1.64; 95% CI, 1.17–2.30).

**Conclusions:**

Preswitch regimens containing efavirenz and TDF were associated with significant weight gain after switching to INSTI-based regimens. Our findings support the hypothesis that the weight gain observed with switching to INSTI-based regimens could be driven by stopping medications with weight-suppressing properties.

## Teaser

Among 750 persons with HIV who switched to INSTI-based regimens, pre-switch regimens containing efavirenz and TDF were associated with higher odds of significant weight gain. Our findings suggest that INSTI-associated weight gain may be driven by stopping medications that suppress weight.

Weight gain following initiation of antiretroviral therapy (ART) in people with HIV is common and in some cases is thought to reflect improvement in general health [1]. However, the prevalence of obesity is rising among persons with HIV (PWH) [1]. For the last decade, integrase inhibitors have been the anchor of contemporary antiretroviral regimens. Given the centrality of agents in this drug class to the treatment of HIV, reports of their association with weight gain in PWH, a group with demonstrable increased risk of cardiovascular disease events, were particularly concerning [2–8].

Recent reports suggest that the observed weight gain among PWH who switch to INSTIs may be associated in part with their preswitch regimen, particularly when they switch off of regimens containing efavirenz or tenofovir disoproxil fumarate (TDF) [9–13]. However, these findings have not yet been widely validated, and other clinical characteristics associated with weight gain when switching to INSTI-based regimens are unclear and consequential to understand given the importance of INSTIs in modern ART. Therefore, to improve our understanding of the role of sequential exposure of antiretroviral agents in weight gain observed among persons on INSTIs, we designed a retrospective study to investigate risk factors for weight gain among PWH who switch to INSTI-based regimens.

## METHODS

### Study Design and Population

We performed an observational, retrospective analysis of all individuals on antiretroviral therapy for the treatment of HIV at the Duke Adult Infectious Diseases Clinic between January 1, 2014, and December 31, 2021, who switched to an INSTI-based regimen. Data were extracted from the electronic health record (EHR). Preswitch and postswitch regimens and their durations up to 24 months were confirmed through manual chart review. Individuals who took the postswitch regimen for <1 month were excluded. Individuals were also excluded if they had missing weight data within 90 days of either the duration of their new regimen or 24 months postswitch, whichever was shorter. This study received approval from the Duke Institutional Review Board.

### Outcomes and Exposures

The outcome in our analyses was weight change operationalized in several ways. Percent weight change was defined as the difference between follow-up and preswitch weight (measured on or before the switch date to a maximum of 24 months) divided by preswitch weight; absolute weight change was defined as the difference between follow-up and preswitch weight. We also created binary indicator variables of whether percent weight change was greater than 5% or 10%. The primary exposure was preswitch regimen, categorized into 7 groups: efavirenz/emtricitabine/tenofovir disoproxil fumarate (EFV/FTC/TDF), cobicistat/elvitegravir/emtricitabine/tenofovir disoproxil fumarate (COBI/EVG/FTC/TDF), cobicistat/elvitegravir/emtricitabine/tenofovir alafenamide (COBI/EVG/FTC/TAF), raltegravir/emtricitabine/tenofovir disoproxil fumarate (RAL/FTC/TDF), any regimen with darunavir/ritonavir (DRV/r), atazanavir/ritonavir (ATV/r), or lopinavir/ritonavir (LPV/r), any other efavirenz-containing regimen, and all other remaining regimens. We also dichotomized preswitch regimen into those containing efavirenz (yes/no) and those containing TDF (yes/no).

Covariates of interest included age at switch date, sex at birth, race, ethnicity, body mass index (BMI), CD4 count, and HIV-1 viral load (VL) at time of switch. Race and ethnicity were included in these analyses due to the effects of systemic racism and discrimination, which could confound associations between preswitch regimen and weight gain [14, 15]. BMI was categorized into <25 kg/m^2^, 25 to <30 kg/m^2^, and ≥30 kg/m^2^.

### Statistical Analysis

We described cohort characteristics using counts and percentages for categorical variables and mean (SD) or median (interquartile range [IQR]) as appropriate for continuous variables. We presented these both overall and by whether ≥5% body weight was gained after regimen switch.

We used separate generalized linear models to assess the association between preswitch regimen and each outcome described above, adjusted for age, sex, race, ethnicity, and preswitch BMI as these could potentially be associated with both preswitch regimen and weight gain. Those with missing covariate data were excluded from these analyses. The continuous outcomes of percent and absolute weight change were modeled with an identity link, while the binary outcomes of ≥5% and ≥10% weight gain were modeled with a logit link. Preswitch regimen was modeled separately as a 7-level multicategorical variable, as a binary variable of efavirenz-containing regimen, and as a binary variable of TDF-containing regimen. Because individuals had different durations of INSTI-based regimens, we performed sensitivity analyses only among those who had been on their postswitch regimen for at least 24 months. Additionally, we conducted separate sensitivity analyses with the following modifications to the analysis cohort: (1) only persons who had achieved viral suppression, (2) only persons who were not exposed to TDF postswitch, and (3) only persons who were not exposed to TAF postswitch.

In our initial results, we noted that many individuals had preswitch regimens containing both efavirenz and TDF. To better ascertain whether TDF-containing preswitch regimens were independently associated with weight gain, we performed a post hoc analysis comparing individuals with a preswitch regimen of EFV/FTC/TDF with those with a preswitch regimen containing TDF and protease inhibitors, which are historically associated with weight gain [16–18].

Analyses were performed using R, version 4.1.3 (R Foundation for Statistical Computing, Vienna, Austria), and all hypothesis tests were 2-sided. The analyses were performed from July 2022 to May 2024.

## RESULTS

### Cohort Characteristics

Our cohort included 750 individuals (Table 1). The mean age (SD) was 51 (11) years, and 30% of individuals were assigned female sex at birth. The mean CD4 count at the time of switch (SD) was 701 (370) cells/mm^3^, and 68% of individuals had an undetectable HIV-1 viral load at medication switch. The median duration of postswitch regimen (IQR) was 24 (19–24) months, with follow-up ranging from 1 to 24 months (Table 1). The mean weight change after regimen switch (SD) was 1.78 (7.03) kg, or 2.4% (8.8%) of preswitch weight. A total of 229 (31%) individuals gained ≥5% of preswitch weight, and 92 (12%) individuals gained ≥10% of preswitch weight.

**Table 1.** Cohort Characteristics

	Overall (n = 750)	Weight Gain ≥5%	
		No (n = 521)	Yes (n = 229)
Age, y	50.9 (11.3)	51.4 (11.1)	49.9 (11.6)
Female sex	222 (29.6%)	143 (27.4%)	79 (34.5%)
Race			
Black or African American	431 (57.5%)	296 (56.8%)	135 (59.0%)
White	259 (34.5%)	183 (35.1%)	76 (33.2%)
Other race^a^	37 (4.9%)	25 (4.8%)	12 (5.2%)
Not reported/declined	23 (3.1%)	17 (3.3%)	6 (2.6%)
Ethnicity			
Not Hispanic	695 (92.7%)	479 (91.9%)	216 (94.3%)
Hispanic	29 (3.9%)	19 (3.6%)	10 (4.4%)
Not reported/declined	26 (3.5%)	23 (4.4%)	3 (1.3%)
CD4, cells/mm^3^	701 (370)	720 (357)	660 (397)
Undetectable viral load (<20)	513 (68.4%)	351 (67.4%)	162 (70.7%)
Viral suppression (<200)	593 (79.1%)	411 (78.9%)	182 (79.5%)
Preswitch BMI, kg/m^2^	29.0 (6.80)	29.5 (6.84)	27.8 (6.54)
Preswitch BMI (categorized)			
<25 kg/m^2^	218 (29.1%)	131 (25.1%)	87 (38.0%)
25 to <30 kg/m^2^	256 (34.1%)	187 (35.9%)	69 (30.1%)
≥30 kg/m^2^	276 (36.8%)	203 (39.0%)	73 (31.9%)
Time on postswitch regimen, median (IQR), mo	24.0 (19.0–24.0)	24.0 (16.0–24.0)	24.0 (24.0–24.0)

Values are reported as mean (SD) or count (%) unless otherwise specified.

^a^Other race includes American Indian or Alaskan Native, Asian, Native Hawaiian or other Pacific Islander, and 2 or more races; these were collapsed to protect identities given small counts.

### Demographics Associated With Weight Gain

In multivariable models with preswitch regimen as a 7-level multicategorical variable, female sex at birth was associated with weight gain. Compared with males, females gained an additional 1.72 kg (95% CI, 0.53–2.91) on average. Females had significantly higher odds of gaining ≥5% of preswitch weight (odds ratio [OR], 1.80; 95% CI, 1.00–3.27) and ≥10% of preswitch weight (OR, 2.71; 95% CI, 1.24–5.88) compared with males.

Persons with a BMI ≥25 kg/m^3^ gained significantly less weight than persons with lower BMIs. In our multivariable analysis, persons with a BMI between 25 and 30 kg/m^3^ gained 1.5 kg less than persons with a BMI <25 kg/m^3^. Persons with a BMI >30 kg/m^3^ gained 2.3 kg less than persons with a BMI <25 kg/m^3^. Odds ratios for gaining ≥10% body weight postswitch (compared with the reference group of BMI <25) were 0.45 (95% CI, 0.20–0.98) for persons with a BMI of 25–30 kg/m^3^ and 0.30 (95% CI, 0.12–0.70) for persons with BMI >30 (Table 2).

**Table 2.** Multivariate Analysis of Weight Change

Variable	% Weight Change Est (95% CI)	Absolute Weight Change Est (95% CI), kg	Gained ≥5% Weight OR (95% CI)^a^	Gained ≥10% Weight OR (95% CI)^a^
Age (per 5 y)	0.17 (−0.27 to 0.62)	0.20 (−0.18 to 0.58)	1.04 (0.93 to 1.18)	0.96 (0.82 to 1.12)
Sex				
Male	(ref)	(ref)	(ref)	(ref)
Female	2.01 (−0.24 to 4.26)	0.86 (−1.05 to 2.77)	**1.80 (1.00 to 3.27)**	**2.71 (1.24 to 5.88)**
Race				
Non-Black	(ref)	(ref)	(ref)	(ref)
Black	1.04 (−0.97 to 3.05)	0.59 (−1.12 to 2.30)	1.06 (0.62 to 1.82)	1.07 (0.50 to 2.28)
Not reported/declined	0.50 (−5.96 to 6.97)	0.80 (−4.70 to 6.30)	3.12 (0.48 to 25.13)	N/A
Ethnicity				
Not Hispanic	(ref)	(ref)	(ref)	(ref)
Hispanic	4.70 (0.73 to 8.66)	2.59 (−0.78 to 5.96)	1.10 (0.35 to 3.10)	2.43 (0.60 to 8.26)
Not reported/declined	−0.96 (−8.60 to 6.68)	−1.52 (−8.01 to 4.97)	N/A	N/A
BMI				
<25 kg/m^2^	(ref)	(ref)	(ref)	(ref)
25 to <30 kg/m^2^	−3.09 (−5.32 to −0.87)	−1.53 (−3.42 to 0.36)	**0.48 (0.26 to 0.85)**	**0.45 (0.20 to 0.98)**
≥30 kg/m^2^	−4.67 (−7.01 to −2.33)	−2.31 (−4.30 to −0.32)	**0.38 (0.20 to 0.72)**	**0.30 (0.12 to 0.70)**
Individual preswitch regimens				
All other regimens	(ref)	(ref)	(ref)	(ref)
EFV/FTC/TDF	**2.93 (0.93 to 4.93)**	**2.45 (0.84 to 4.06)**	**1.83 (1.09 to 3.1)**	**2.47 (1.16 to 5.59)**
COBI/EVG/FTC/TDF	**3.35 (0.55 to 6.14)**	**3.21 (0.96 to 5.46)**	**2.72 (1.36 to 5.47)**	2.38 (0.85 to 6.51)
COBI/EVG/FTC/TAF	0.03 (−2.13 to 2.19)	0.19 (−1.55 to 1.92)	0.91 (0.5 to 1.65)	1.23 (0.49 to 3.05)
RAL/FTC/TDF	1.42 (−1.36 to 4.19)	1.01 (−1.22 to 3.25)	1.54 (0.75 to 3.12)	1.7 (0.57 to 4.75)
Regimen with DRV/r, ATV/r, or LPV/r	0.09 (−1.84 to 2.01)	0.21 (−1.34 to 1.76)	1.11 (0.66 to 1.87)	1.68 (0.79 to 3.76)
Other EFV regimen	**4.16 (0.61 to 7.72)**	**3.34 (0.48 to 6.2)**	**2.6 (1.08 to 6.22)**	2.4 (0.68 to 7.64)
Preswitch EFV				
No	(ref)	(ref)	(ref)	(ref)
Yes	**2.62 (1.19 to 4.05)**	**2.06 (0.9 to 3.21)**	**1.62 (1.13 to 2.32)**	**1.68 (1.02 to 2.73)**
Preswitch TDF				
No	(ref)	(ref)	(ref)	(ref)
Yes	**1.33 (0.04 to 2.62)**	**1.12 (0.08 to 2.16)**	**1.64 (1.17 to 2.3)**	1.39 (0.87 to 2.26)

Statistically significant results, in which confidence intervals exclude the null, are bolded.

Abbreviations: ATV/r, atazanavir/ritonavir; BMI, body mass index; COBI, cobicistat; DRV/r, darunavir/ritonavir; EFV, efavirenz; EVG, elvitegravir; FTC, emtricitabine; LPV/r, lopinavir/ritonavir; OR, adjusted odds ratio; RAL, raltegravir; TAF, tenofovir alafenamide; TDF, tenofovir disoproxil fumarate.

^a^Outcomes are adjusted for age, sex, race, ethnicity, and preswitch BMI.

### Preswitch Regimens Associated With Weight Gain

The most common preswitch regimens were EFV/FTC/TDF (Atripla; n = 170, 22.7%), COBI/EVG/FTC/TAF (Genvoya; n = 121, 16.1%), COBI/EVG/FTC/TDF (Stribild; n = 58, 7.7%), and RAL/FTC/TDF (n = 54, 7.2%). A total of 194 (25.9%) individuals had a preswitch regimen containing efavirenz; of these, 174 (89.7%) also contained TDF (Supplementary Tables 1–3). A total of 452 (60.3%) individuals had a preswitch regimen containing TDF; of these, 174 (38.5%) also contained efavirenz (Supplementary Tables 1–3).

**Figure 1.**
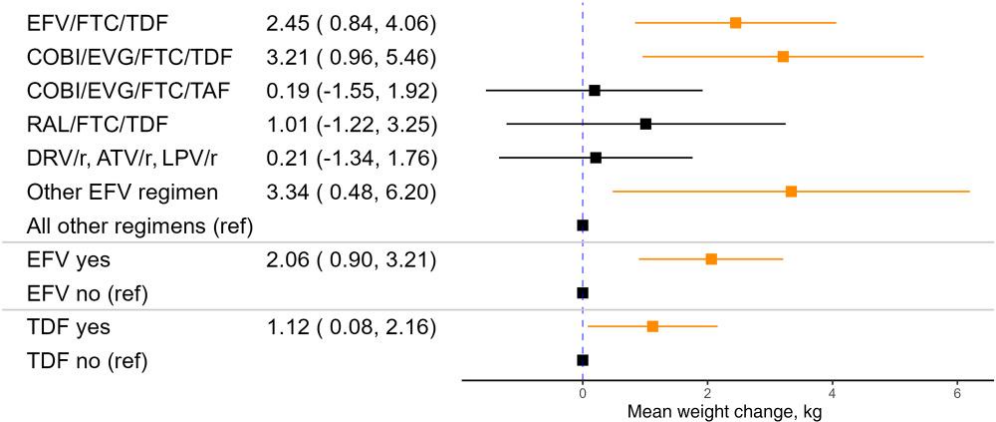
Mean percent weight change after regimen switch by preswitch regimen. Adjusted for age, sex, race, ethnicity, and preswitch BMI. Abbreviations: ATV/r, atazanavir/ritonavir; BMI, body mass index; COBI, cobicistat; DRV/r, darunavir/ritonavir; EFV, efavirenz; EVG, elvitegravir; FTC, emtricitabine; LPV/r, lopinavir/ritonavir; RAL, raltegravir; TAF, tenofovir alafenamide; TDF, tenofovir disoproxil fumarate.

**Figure 2.**
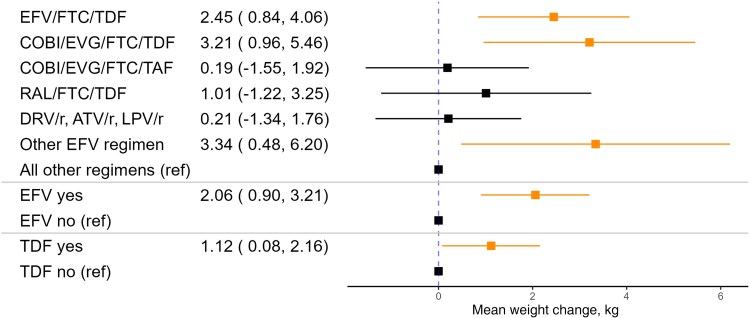
Mean absolute weight change in kilograms after regimen switch by preswitch regimen. Adjusted for age, sex, race, ethnicity, and preswitch BMI. Abbreviations: ATV/r, atazanavir/ritonavir; BMI, body mass index; COBI, cobicistat; DRV/r, darunavir/ritonavir; EFV, efavirenz; EVG, elvitegravir; FTC, emtricitabine; LPV/r, lopinavir/ritonavir; RAL, raltegravir; TAF, tenofovir alafenamide; TDF, tenofovir disoproxil fumarate.

**Figure 3.**
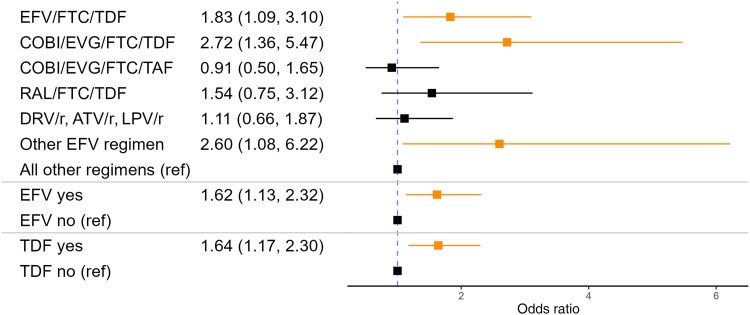
Odds ratios of gaining ≥5% body weight after regimen switch by preswitch regimen. Adjusted for age, sex, race, ethnicity, and preswitch BMI. Abbreviations: ATV/r, atazanavir/ritonavir; BMI, body mass index; COBI, cobicistat; DRV/r, darunavir/ritonavir; EFV, efavirenz; EVG, elvitegravir; FTC, emtricitabine; LPV/r, lopinavir/ritonavir; RAL, raltegravir; TAF, tenofovir alafenamide; TDF, tenofovir disoproxil fumarate.

**Figure 4.**
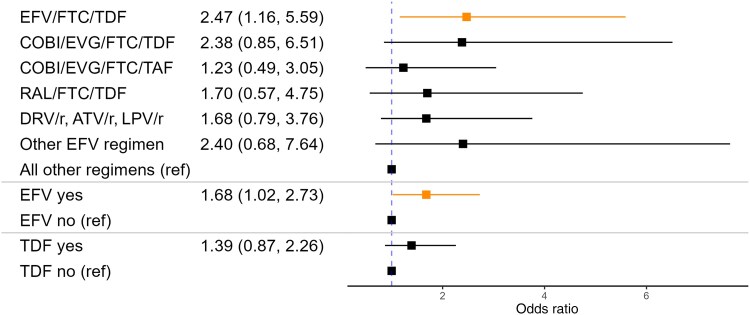
Odds ratios of gaining ≥10% body weight after regimen switch by preswitch regimen. Adjusted for age, sex, race, ethnicity, and preswitch BMI. Abbreviations: ATV/r, atazanavir/ritonavir; BMI, body mass index; COBI, cobicistat; DRV/r, darunavir/ritonavir; EFV, efavirenz; EVG, elvitegravir; FTC, emtricitabine; LPV/r, lopinavir/ritonavir; RAL, raltegravir; TAF, tenofovir alafenamide; TDF, tenofovir disoproxil fumarate.

In multivariable models with dichotomized preswitch regimens, individuals with any preswitch regimen containing efavirenz gained an average of 2.62% (95% CI, 1.19%–4.05%) more of their body weight after regimen switch than those with other preswitch regimens (Figure 1), corresponding to an average of 2.06 additional kg gained (95% CI, 0.9–3.21) (Figure 2). Individuals with efavirenz-containing preswitch regimens had higher odds of gaining ≥5% body weight (OR, 1.62; 95% CI, 1.13–2.32) (Figure 3) and ≥10% body weight (OR, 1.68; 95% CI, 1.02–2.73) (Figure 4).

Similarly, preswitch regimens containing TDF were significantly associated with weight gain. Individuals with any preswitch regimen containing TDF gained an average of 1.33% (95% CI, 0.04%–2.62%) more of their body weight after regimen switch than those with other preswitch regimens (Figure 1), corresponding to an average of 1.12 additional kg gained (95% CI, 0.08–2.16) (Figure 2). Individuals with TDF-containing preswitch regimens had two-thirds higher odds of gaining ≥5% body weight compared with those with other preswitch regimens (OR, 1.64; 95% CI, 1.17–2.3) (Figure 3).

Specific preswitch regimens associated with significant weight gain included EFV/FTC/TDF (Atripla) and COBI/EVG/FTC/TDF (Stribild). Individuals with these preswitch regimens gained an average of 2.93% (95% CI, 0.93%–4.93%) and 3.35% (95% CI, 0.55%–6.14%) more of their preswitch body weight, respectively (Figure 1). These findings persisted when the analysis cohort was only restricted to persons who were virally suppressed at the time of switch (Table 3).

**Table 3.** Weight Gain After Regimen Switch by Preswitch Regimen Among Persons With Viral Suppression at Time of Switch (n = 592)

Variable	% Weight Change Est (95% CI)	Absolute Weight Change Est (95% CI), kg	Gained ≥5% Weight OR (95% CI)^a^	Gained ≥10% Weight OR (95% CI)^a^
Preswitch regimen				
All other regimens^b^	(ref)	(ref)	(ref)	(ref)
EFV/FTC/TDF	**3.89 (1.87 to 5.91)**	**3.04 (1.31 to 4.76)**	**1.94 (1.1 to 3.51)**	**4.29 (1.64 to 13.5)**
COBI/EVG/FTC/TDF	**4.42 (1.56 to 7.28)**	**3.97 (1.52 to 6.42)**	**3.04 (1.4 to 6.71)**	3.42 (0.92 to 13.21)
COBI/EVG/FTC/TAF	0.44 (−1.7 to 2.59)	0.33 (−1.51 to 2.16)	0.76 (0.39 to 1.49)	1.13 (0.32 to 4.1)
RAL/FTC/TDF	2.49 (−0.38 to 5.35)	1.53 (−0.93 to 3.98)	1.49 (0.65 to 3.33)	2.99 (0.82 to 11.39)
Regimen with DRV/r, ATV/r, or LPV/r	1.86 (−0.14 to 3.86)	1.51 (−0.2 to 3.22)	1.22 (0.68 to 2.22)	**3.39 (1.29 to 10.67)**
Other EFV regimen	**4.92 (1.34 to 8.5)**	**3.60 (0.54 to 6.67)**	**2.86 (1.08 to 7.59)**	3.98 (0.88 to 17.05)
Preswitch EFV				
No	(ref)	(ref)	(ref)	(ref)
Yes	**2.68 (1.23 to 4.12)**	**2.03 (0.79 to 3.27)**	**1.73 (1.17 to 2.57)**	**1.97 (1.12 to 3.42)**
Preswitch TDF				
No	(ref)	(ref)	(ref)	(ref)
Yes	**2.07 (0.75 to 3.38)**	**1.72 (0.6 to 2.85)**	**1.81 (1.24 to 2.65)**	**1.82 (1.05 to 3.28)**

Statistically significant results, in which confidence intervals exclude the null, are bolded.

Abbreviations: ATV/r, atazanavir/ritonavir; BMI, body mass index; COBI, cobicistat; DRV/r, darunavir/ritonavir; EFV, efavirenz; EVG, elvitegravir; FTC, emtricitabine; LPV/r, lopinavir/ritonavir; OR, adjusted odds ratio; RAL, raltegravir; TAF, tenofovir alafenamide; TDF, tenofovir disoproxil fumarate.

^a^Outcomes are adjusted for age, sex, race, ethnicity, and preswitch BMI.

^b^See Supplementary Table 2 for a complete list of preswitch regimens.

Our findings that preswitch regimens containing efavirenz and TDF were associated with weight gain were consistent in sensitivity analyses limited to individuals who completed 24 months of follow-up (n = 515, 68.7% of total) (Supplementary Table 4). In this analysis, individuals taking a preswitch regimen of RAL/FTC/TDF were also found to have higher odds of gaining ≥5% body weight after regimen switch (OR, 2.72; 95% CI, 1.10–6.86).

In a post hoc sensitivity analysis, compared with individuals taking a preswitch regimen containing TDF and a protease inhibitor (n = 161), we found that individuals taking a preswitch regimen of EFV/FTC/TDF (n = 166) gained more weight on average (2.98 ± 6.67 vs 1.23 ± 7.19 kg; *P* = .024) (Supplementary Table 5). However, the odds of ≥5% or ≥10% weight gain did not significantly differ between groups.

We also conducted separate sensitivity analyses excluding persons with postswitch TDF exposure (Supplementary Table 6) and postswitch TAF exposure (Supplementary Table 7), given the reported association of these agents with appetite suppression and weight gain, respectively. In the sensitivity analysis excluding persons with postswitch TDF exposure, the association with preswitch EFV exposure and >10% weight gain postswitch no longer met statistical significance (OR, 1.58; 95% CI, 0.93–2.63). However, similar to the primary analysis, EFV preswitch exposure remained independently associated with absolute weight gain and events of ≥5% weight gain post–switch to INSTIs. In the sensitivity analysis excluding persons with postswitch TAF exposure, we found that although the absolute weight change postswitch associated with a preswitch regimen of EFV/FTC/TDF remained significant (2.84 kg; 95% CI, 0.69–4.99 kg), its association with odds of 5% weight gain did not reach statistical significance (OR, 1.98; 95% CI, 0.99–4.06).

## DISCUSSION

We found that preswitch regimens containing efavirenz and TDF were associated with higher odds of significant weight gain after switching to regimens with second-generation INSTIs. Specific preswitch regimens associated with subsequent weight gain included EFV/FTC/TDF (Atripla) and COBI/EVG/FTC/TDF (Stribild). These results support the hypothesis that the weight gain observed with switching to INSTI-based regimens may be driven by stopping certain ARTs associated with weight suppression, rather than weight gain directly caused by INSTIs.

The association between efavirenz and weight suppression is supported by recent pharmacogenetic studies. Efavirenz is metabolized by CYP2B6, resulting in higher plasma concentrations of efavirenz among *CYP2B6* slow and intermediate metabolizers. In a cohort study of individuals switching from efavirenz- to INSTI-containing regimens, *CYP2B6* slow metabolizers gained more weight after regimen switch [9]. Similarly, in a subset analysis of the ADVANCE trial, *CYP2B6* genotype was strongly associated with weight gain among individuals starting an efavirenz-based regimen [10]. The mechanism of weight loss with efavirenz is unclear but may be related to known neuropsychiatric effects that could affect appetite [19].

Multiple recent studies have also linked TDF with weight suppression. A review of 7 pre-exposure prophylaxis (PrEP) trials found that HIV-negative individuals taking TDF were more likely to experience weight loss [20]. In a Kenyan study of PWH switching from NNRTI-based regimens to a DTG-based regimen, participants with a preswitch regimen of TDF/3TC had greater increases in the rate of weight gain compared with the overall population [21]. A German cohort study noted lower risk of weight gain among PWH with TDF-containing regimens [22]. In the CHARACTERISE trial in South Africa, women who switched from TAF/FTC/DTG to TDF/3TC/DTG showed a significant decrease in body weight, losing a median of 1.6 kg [23]. Finally, in the ADVANCE trial, patients who started ART with DTG had less weight gain when the regimen also included TDF [2].

Our study found that female individuals had higher odds of gaining weight after switching to INSTI-based regimens. This finding is consistent with the ADVANCE trial, which found that female individuals gained more weight after starting ART, including both INSTI-based regimens and a standard-of-care regimen [2]. We also found that persons with a low BMI (≤20) had higher odds of significant weight gain after switching to INSTI-based regimens. Notably, the association between preswitch regimens containing efavirenz and TDF and weight gain were significant when adjusted for sex and preswitch BMI. This finding is contrary to prior reports that showed that persons with BMI >30 were more likely to gain significant weight after switch to INSTI. We also did not observe the increased weight gain associated with Black race or increased age at switch in our analysis as demonstrated in prior reports [12].

Because many individuals took preswitch regimens containing both efavirenz and TDF, the relative effects of each medication on subsequent weight gain were difficult to delineate. However, both preswitch efavirenz and TDF appeared to be independently associated with subsequent weight gain after regimen switch. Of the 2 specific preswitch regimens associated with weight gain after regimen switch, 1 was a regimen containing TDF without efavirenz (COBI/EVG/FTC/TDF). Individuals with analogous preswitch regimens containing TAF rather than TDF did not have significant weight gain after regimen switch. We were unable to meaningfully compare weight changes between individuals with preswitch regimens containing efavirenz with or without TDF, as only 20 individuals had a preswitch regimen containing efavirenz without TDF. However, in our post hoc analysis, we found that individuals on a preswitch regimen of EFV/FTC/TDF had an average of about 2 kg greater weight gain after regimen switch than those with a preswitch regimen containing TDF and a protease inhibitor. This suggests that preswitch efavirenz may have driven increased weight gain among these individuals.

Our study has several limitations. First, our study participants were limited to a single HIV clinic, which limited sample size and may limit applicability to other sites. However, unlike previous reports investigating weight gain after switching to an INSTI-based regimen, not all our participants were virally suppressed, which may increase the applicability of our findings to real-world clinics. Reason for ART switch could not be ascertained in our manual chart review. In addition, weight change outcomes in our primary analysis did not account for time between pre- and postswitch weight measurements or duration of postswitch regimen. To address this, however, we performed sensitivity analyses in which all individuals were on the INSTI-based postswitch regimen for 24 months. Lastly, as with all observational studies, effects of unmeasured confounding likely exist and limit our ability to draw causal inference. This includes potential effects of weight-altering concomitant medications such as GLP-1 agonist or hormone therapy that were beyond the scope of this study, comorbidities, and ART adherence.

In conclusion, our findings may inform clinicians' decision-making of whether to switch patients on ART to INSTI-based regimens. Clinicians should be aware that significant weight gain may be driven by preswitch regimens containing efavirenz or TDF, and women and underweight individuals may be most susceptible to weight gain. Further studies are needed to validate these findings in a more diverse patient population and explore additional risk factors for weight gain after switching to INSTI-based regimens.

## Supplementary Data

Supplementary materials are available at *Open Forum Infectious Diseases* online. Consisting of data provided by the authors to benefit the reader, the posted materials are not copyedited and are the sole responsibility of the authors, so questions or comments should be addressed to the corresponding author.

## Supplementary Material

ofae752_Supplementary_Data

## Note

***Financial support*.** This work was supported by the National Institutes of Health (K23 HL137611) and the Duke Center for AIDS Research (P30 AI064518).
