# Supplementary material for: Preswitch Regimens Associated With Weight Gain Among Persons With HIV who Switch to Integrase Inhibitor–Containing Regimens
Source: Open Forum Infect Dis. 2025 Mar 21;12(3):ofae752. doi: 10.1093/ofid/ofae752 (PMC11927775; doi:10.1093/ofid/ofae752)
Supplement: ofae752_Supplementary_Data [file ofae752_supplementary_data.docx]

**Supplement**

**Supplementary Table 1. Cohort characteristics by pre-switch regimen.** Values are reported as count (%).

|  | **Overall** (N=750) | **Weight gain ≥5%** | |
| --- | --- | --- | --- |
|  |  | **No** (N=521) | **Yes** (N=229) |
| **Pre-switch regimen**  All other regimens**  EFV/FTC/TDF | 133 (17.7%)  166 (22.1%) | 99 (19.0%)  107 (20.5%) | 34 (14.8%)  59 (25.8%) |
| COBI/EVG/FTC/TDF  COBI/EVG/FTC/TAF | 55 (7.3%)  120 (16.0%) | 29 (5.6%)  93 (17.9%) | 26 (11.4%)  27 (11.8%) |
| RAL/FTC/TDF | 54 (7.2%) | 36 (6.9%) | 18 (7.9%) |
| Regimen with DRV/r, ATV/r or LPV/r | 194 (25.9%) | 142 (27.3%) | 52 (22.7%) |
| Other EFV regimen | 28 (3.7%) | 15 (2.9%) | 13 (5.7%) |
| **Pre-switch EFV**  **Pre-switch TDF** | 194 (25.9%)  452 (60.3%) | 122 (23.4%)  296 (56.8%) | 72 (31.4%)  156 (68.1%) |

**Supplementary Table 2.** Complete list of pre-switch ART regimens

| Pre-switch regimen | n |
| --- | --- |
| EFV_FTC_TDF | 170 |
| COBI_EVG_FTC_TAF | 121 |
| COBI_EVG_FTC_TDF | 58 |
| FTC_RAL_TDF | 54 |
| ATV/r_FTC_TDF | 41 |
| DRV/r_FTC_TDF | 35 |
| FTC_RPV_TDF | 19 |
| 3TC_ABC_RAL | 12 |
| DRV/r_RAL | 12 |
| FTC_LPV/r_TDF | 11 |
| 3TC_ABC_ATV/r | 9 |
| 3TC_ABC_EFV | 9 |
| FTC_RAL_TAF | 9 |
| COBI_DRV_FTC_TAF | 8 |
| DRV/r_ETR_RAL | 7 |
| DRV/r_FTC_RAL_TDF | 7 |
| 3TC_ABC_LPV/r | 5 |
| 3TC_AZT_EFV | 5 |
| 3TC_AZT_NVP | 5 |
| ATV/r_FTC_RAL_TDF | 5 |
| COBI_DRV_FTC_TDF | 5 |
| FTC_LPV/r_RAL_TDF | 5 |
| 3TC_ABC_DRV/r | 4 |
| 3TC_ABC_NVP | 4 |
| 3TC_AZT_RAL | 4 |
| DRV/r_ETR | 4 |
| DRV/r_FTC_RPV_TDF | 4 |
| FTC_NVP_TDF | 4 |
| FTC_RPV_TAF | 4 |
| 3TC_ABC_LPV/r_RAL | 3 |
| 3TC_AZT_DRV/r | 3 |
| 3TC_EFV_RAL | 3 |
| ATV/r_RAL | 3 |
| ATV_COBI_FTC_TAF | 3 |
| FPV_FTC_TDF | 3 |
| 3TC_ABC_ATV/r_TDF | 2 |
| 3TC_ABC_AZT_RAL | 2 |
| 3TC_ABC_COBI_DRV | 2 |
| 3TC_ATV/r_AZT | 2 |
| 3TC_AZT_NFV | 2 |
| 3TC_DRV/r_TDF | 2 |
| ABC_EFV_FTC_TDF | 2 |
| ATV/r_EFV | 2 |
| ATV/r_EFV_FTC_TDF | 2 |
| ATV_COBI_FTC_TDF | 2 |
| ATV_FTC_RTV_TDF | 2 |
| COBI_DRV_EVG_FTC_TAF | 2 |
| DRV/r_FTC_TAF | 2 |
| ETR_FTC_RAL_TDF | 2 |
| FTC_TAF | 2 |
| LPV/r_RAL | 2 |
| LPV/r_SQV | 2 |
| 3TC_ABC_ATV | 1 |
| 3TC_ABC_ATV/r_RAL | 1 |
| 3TC_ABC_ATV_COBI | 1 |
| 3TC_ABC_AZT_FPV | 1 |
| 3TC_ABC_AZT_NVP | 1 |
| 3TC_ABC_DLV | 1 |
| 3TC_ABC_DRV/r_ETR | 1 |
| 3TC_ABC_FPV/r | 1 |
| 3TC_ABC_LPV/r_NVP | 1 |
| 3TC_ABC_RAL_RPV | 1 |
| 3TC_ABC_RPV | 1 |
| 3TC_ABC_SQV/r | 1 |
| 3TC_ATV/r_AZT_RAL | 1 |
| 3TC_ATV_DDI | 1 |
| 3TC_ATV_NVP | 1 |
| 3TC_AZT_COBI_DRV | 1 |
| 3TC_AZT_DRV/r_ETR | 1 |
| 3TC_AZT_LPV/r_TDF | 1 |
| 3TC_DRV/r_ETR | 1 |
| 3TC_DRV/r_RAL | 1 |
| 3TC_EFV_TDF | 1 |
| 3TC_FPV/r | 1 |
| 3TC_FPV/r_RAL | 1 |
| 3TC_NVP_RAL | 1 |
| 3TC_RAL | 1 |
| 3TC_RAL_RPV | 1 |
| ABC_ATV/r_DDI | 1 |
| ABC_ATV/r_FTC_TDF | 1 |
| ABC_ATV/r_TDF | 1 |
| ABC_DRV/r_ETR_LMV_ZDV | 1 |
| ABC_ETR_RAL | 1 |
| ABC_FTC_LPV/r | 1 |
| ABC_FTC_RPV_TDF | 1 |
| ABC_LPV/r_TDF | 1 |
| ABC_TDF_TPV/r | 1 |
| ATV/r_DDI_TDF | 1 |
| ATV/r_ETR_FTC_TAF | 1 |
| ATV/r_ETR_RAL | 1 |
| ATV/r_FTC_TAF | 1 |
| ATV_COBI_DRV/r_FTC_TDF | 1 |
| ATV_COBI_TDF | 1 |
| ATV_FTC_TDF | 1 |
| COBI_DRV/r_RAL | 1 |
| COBI_DRV_ETR | 1 |
| COBI_DRV_ETR_FTC_TDF | 1 |
| COBI_DRV_EVG_FTC_TDF | 1 |
| COBI_DRV_RAL | 1 |
| DDL_EFV_TDF | 1 |
| DRV/r_EFV | 1 |
| DRV/r_ETR_FTC_TAF | 1 |
| DRV/r_ETR_FTC_TDF | 1 |
| DRV/r_FTC_RAL | 1 |
| DRV/r_FTC_RAL_TAF | 1 |
| DRV/r_FTC_RPV_TAF | 1 |
| DRV/r_FTC_RTV_TDF | 1 |
| DRV/r_NVP_TDF | 1 |
| DRV_ETR_RAL_RTV | 1 |
| DRV_FTC_TDF | 1 |
| EFV/r_FTC_SQV_TDF | 1 |
| ETR_FTC_RAL_TAF | 1 |
| ETR_FTC_TDF | 1 |
| FPV_FTC_RAL/r_TDF | 1 |
| FPV_FTC_RAL_TDF | 1 |
| FTC_NVP_RAL_TDF | 1 |
| FTC_NVP_TAF | 1 |
| FTC_RAL/r_TDF | 1 |
| FTC_RAL_RPV_TAF | 1 |
| FTC_RAL_RPV_TDF | 1 |
| LPV/r | 1 |
| LPV/r_NVP | 1 |
| MVC_RAL_TDF | 1 |

**Supplementary Table 3.** Complete list of post-switch ART regimens

| Post-switch regimen | n |
| --- | --- |
| BIC_FTC_TAF | 241 |
| 3TC_ABC_DTG | 226 |
| DTG_FTC_TDF | 80 |
| DTG_FTC_TAF | 43 |
| COBI_DRV_DTG | 26 |
| COBI_DRV_DTG_FTC_TDF | 12 |
| COBI_DRV_DTG_FTC_TAF | 11 |
| DRV/r_DTG | 11 |
| DTG_RPV | 11 |
| 3TC_DTG | 9 |
| DRV/r_DTG_FTC_TDF | 9 |
| DTG_FTC_RPV_TDF | 8 |
| 3TC_DRV/r_DTG | 5 |
| DRV/r_DTG_ETR | 5 |
| 3TC_ABC_DTG_TDF | 4 |
| 3TC_AZT_DTG | 4 |
| 3TC_ABC_DRV/r_DTG | 3 |
| 3TC_DTG_RPV | 3 |
| COBI_DRV_DTG_RPV | 3 |
| 3TC_COBI_DRV_DTG | 2 |
| 3TC_DTG_TDF | 2 |
| ATV/r_DTG_FTC_TAF | 2 |
| ATV/r_DTG_FTC_TDF | 2 |
| ATV_COBI_DTG | 2 |
| COBI_DRV_DTG_ETR | 2 |
| DRV/r_DTG_RPV | 2 |
| DTG_FTC | 2 |
| 3TC_ABC_ATV/r_DTG | 1 |
| 3TC_ABC_COBI_DRV_DTG | 1 |
| 3TC_ABC_DTG_LPV/r | 1 |
| 3TC_AZT_DTG_RPV | 1 |
| 3TC_DOR_DTG | 1 |
| 3TC_DTG_EFV | 1 |

**Supplementary Table 4. Sensitivity analysis of weight gain after regimen switch among patients with 24 months of follow up (n=515).** EFV: efavirenz. FTC: emtricitabine. TDF: tenofovir disoproxil fumarate. COBI: cobicistat. EVG: elvitegravir. TAF: tenofovir alafenamide. RAL: raltegravir. DRV/r: darunavir/ritonavir. ATV/r: atazanavir/ritonavir. LPV/r: lopinavir/ritonavir. CI: confidence interval. OR: adjusted odds ratio.

| Variable | % weight change  Est (95% CI) | Absolute weight change in kg  Est (95% CI) | Gained ≥5% weight  OR (95% CI)* | Gained ≥10% weight  OR (95% CI)* |
| --- | --- | --- | --- | --- |
| Pre-switch regimen |  |  |  |  |
| All other regimens | (ref) | (ref) | (ref) | (ref) |
| EFV/FTC/TDF | 2.41 (-0.05, 4.87) | **2.41 (0.42, 4.39)** | 1.74 (0.94, 3.27) | **2.53 (1.02, 6.97)** |
| COBI/EVG/FTC/TDF | 2.43 (-0.87, 5.74) | **2.84 (0.17, 5.51)** | **2.32 (1.04, 5.26)** | 2.46 (0.77, 8.02) |
| COBI/EVG/FTC/TAF | -0.89 (-3.57, 1.79) | -0.02 (-2.19, 2.14) | 0.91 (0.45, 1.83) | 1.29 (0.45, 3.91) |
| RAL/FTC/TDF | 1.98 (-1.77, 5.72) | 1.79 (-1.24, 4.82) | **2.72 (1.1, 6.86)** | 2.62 (0.73, 9.14) |
| Regimen with DRV/r, ATV/r or LPV/r | -0.35 (-2.77, 2.07) | 0.28 (-1.67, 2.24) | 1 (0.54, 1.9) | 1.66 (0.66, 4.6) |
| Other EFV regimen | 3.66 (-0.25, 7.57) | 3.04 (-0.12, 6.2) | **2.65 (1.03, 6.89)** | 2.25 (0.52, 8.67) |
| Pre-Switch EFV |  |  |  |  |
| No | (ref) | (ref) | (ref) | (ref) |
| Yes | **2.53 (0.86, 4.21)** | **1.97 (0.61, 3.32)** | **1.58 (1.05, 2.38)** | 1.59 (0.9, 2.78) |
| Pre-switch TDF |  |  |  |  |
| No | (ref) | (ref) | (ref) | (ref) |
| Yes | 1.52 (-0.06, 3.11) | **1.3 (0.02, 2.58)** | **1.59 (1.07, 2.38)** | 1.44 (0.83, 2.56) |

*Outcomes are adjusted for age, sex, race, ethnicity, and pre-switch BMI.

**Supplementary Table 5. Post-hoc analysis of weight gain after regimen switch by pre-switch regimen of EFV/FTC/TDF (n=166) versus pre-switch regimens containing TDF and a protease inhibitor (n=161).** Models are adjusted for age, sex, race, ethnicity, and pre-switch BMI. EFV: efavirenz. FTC: emtricitabine. TDF: tenofovir disoproxil fumarate. PI: protease inhibitor.

|  | Overall (N=327) | TDF+PI (N=161) | EFV/FTC/TDF (N=166) | P-value |
| --- | --- | --- | --- | --- |
| **Percent weight change** |  |  |  | **0.029** |
| Mean (SD) | 2.88 (8.36) | 1.86 (8.42) | 3.87 (8.21) |  |
| Median [Q1, Q3] | 2.92 [-1.25, 6.31] | 2.21 [-2.15, 5.33] | 3.36 [-0.64, 7.18] |  |
| **Absolute weight change (kg)** |  |  |  | **0.024** |
| Mean (SD) | 2.12 (6.97) | 1.23 (7.19) | 2.98 (6.67) |  |
| Median [Q1, Q3] | 2.27 [-1.25, 5.20] | 1.81 [-1.91, 4.17] | 2.72 [-0.447, 5.78] |  |
| **Gained** ≥**5% weight** |  |  |  | 0.46 |
| No | 218 (66.7) | 111 (68.9) | 107 (64.5) |  |
| Yes | 109 (33.3) | 50 (31.1) | 59 (35.5) |  |
| **Gained** ≥**10% weight** |  |  |  | 0.48 |
| No | 283 (86.5) | 142 (88.2) | 141 (84.9) |  |
| Yes | 44 (13.5) | 19 (11.8) | 25 (15.1) |  |

**Supplementary Table 6. Weight gain by pre-switch regimen after exclusion of persons on tenofovir disoproxil fumarate post-switch (n = 623).** EFV: efavirenz. FTC: emtricitabine. TDF: tenofovir disoproxil fumarate. COBI: cobicistat. EVG: elvitegravir. TAF: tenofovir alafenamide. RAL: raltegravir. DRV/r: darunavir/ritonavir. ATV/r: atazanavir/ritonavir. LPV/r: lopinavir/ritonavir. CI: confidence interval. OR: adjusted odds ratio.`

| Variable | % weight change  Est (95% CI) | Absolute weight change in kg  Est (95% CI) | Gained ≥5% weight  OR (95% CI)* | Gained ≥10% weight  OR (95% CI)* |
| --- | --- | --- | --- | --- |
| Pre-switch regimen |  |  |  |  |
| All other regimens | (ref) | (ref) | (ref) | (ref) |
| EFV/FTC/TDF | **2.88 (0.69, 5.07)** | **2.47 (0.71, 4.24)** | **2.35 (1.33, 4.25)** | **2.42 (1.09, 5.72)** |
| COBI/EVG/FTC/TDF | **4.44 (1.33, 7.54)** | **4.22 (1.72, 6.72)** | **4.66 (2.18, 10.23)** | 2.71 (0.95, 7.68) |
| COBI/EVG/FTC/TAF | -0.48 (-2.78, 1.82) | -0.09 (-1.94, 1.76) | 1.1 (0.58, 2.10) | 1.04 (0.40, 2.70) |
| RAL/FTC/TDF | 2.11 (-1.32, 5.54) | 1.51 (-1.25, 4.28) | **2.67 (1.13, 6.27)** | 2.28 (0.69, 7.05) |
| Regimen with DRV/r, ATV/r or LPV/r | -0.01 (-2.20, 2.17) | 0.23 (-1.53, 1.99) | 1.24 (0.68, 2.27) | 1.77 (0.79, 4.21) |
| Other EFV regimen | **4.48 (0.62, 8.34)** | **3.75 (0.64, 6.86)** | **3.12 (1.2, 8.04)** | 2 (0.49, 6.93) |
| Pre-Switch EFV |  |  |  |  |
| No | (ref) | (ref) | (ref) | (ref) |
| Yes | **2.67 (1.09, 4.25)** | **2.09 (0.82, 3.37)** | **1.73 (1.18, 2.54)** | 1.58 (0.93, 2.63) |
| Pre-switch TDF |  |  |  |  |
| No | (ref) | (ref) | (ref) | (ref) |
| Yes | **2.00 (0.58, 3.43)** | **1.64 (0.49, 2.79)** | **1.76 (1.23, 2.53)** | 1.63 (0.99, 2.71) |

**Supplementary Table 7. Weight gain by pre-switch regimen after exclusion of persons on post-switch tenofovir alafenamide (n = 446).** EFV: efavirenz. FTC: emtricitabine. TDF: tenofovir disoproxil fumarate. COBI: cobicistat. EVG: elvitegravir. TAF: tenofovir alafenamide. RAL: raltegravir. DRV/r: darunavir/ritonavir. ATV/r: atazanavir/ritonavir. LPV/r: lopinavir/ritonavir. CI: confidence interval. OR: adjusted odds ratio.`

| Variable | % weight change  Est (95% CI) | Absolute weight change in kg  Est (95% CI) | Gained ≥5% weight  OR (95% CI)* | Gained ≥10% weight  OR (95% CI)* |
| --- | --- | --- | --- | --- |
| Pre-switch regimen |  |  |  |  |
| All other regimens | (ref) | (ref) | (ref) | (ref) |
| EFV/FTC/TDF | **3.01 (0.21, 5.81)** | **2.84 (0.69, 4.99)** | 1.98 (0.99, 4.06) | **3.64 (1.22, 12.61)** |
| COBI/EVG/FTC/TDF | 3.2 (-0.45, 6.84) | **3.37 (0.58, 6.16)** | 2.1 (0.86, 5.12) | 3.78 (0.97, 15.47) |
| COBI/EVG/FTC/TAF | 4.86 (-0.27, 9.98) | 3.85 (-0.07, 7.78) | 2.17 (0.58, 7.42) | 4.39 (0.55, 25.38) |
| RAL/FTC/TDF | 0.57 (-3.09, 4.23) | 0.72 (-2.08, 3.53) | 1.04 (0.38, 2.71) | 1.98 (0.37, 9.28) |
| Regimen with DRV/r, ATV/r or LPV/r | 0.44 (-2.05, 2.93) | 0.82 (-1.09, 2.73) | 1.15 (0.61, 2.23) | 2.29 (0.82, 7.57) |
| Other EFV regimen | **5.52 (1.4, 9.64)** | **4.62 (1.46, 7.77)** | **3.59 (1.34, 9.86)** | **4.7 (1.11, 20.15)** |
| Pre-Switch EFV |  |  |  |  |
| No | (ref) | (ref) | (ref) | (ref) |
| Yes | **2.71 (0.75, 4.67)** | **2.2 (0.7, 3.7)** | **1.85 (1.15, 2.96)** | 1.85 (0.95, 3.55) |
| Pre-switch TDF |  |  |  |  |
| No | (ref) | (ref) | (ref) | (ref) |
| Yes | 0.15 (-1.72, 2.01) | 0.31 (-1.13, 1.74) | 1.31 (0.83, 2.1) | 1.24 (0.64, 2.5) |
